# Supplementary material for: Are carbon emissions trading and green financial instruments synergistic? -Comprehensive quantitative research based on content analysis
Source: PLoS One. 2024 Mar 7;19(3):e0298601. doi: 10.1371/journal.pone.0298601 (PMC10919601; doi:10.1371/journal.pone.0298601)
Supplement: S1 Data — (ZIP) [file pone.0298601.s001.zip › Data/地级市Z_F工作报告.zip!/╣π╬≈/╣≤╕█╩╨/└┤╫╘╣1⁄2╓┌║┼í╛┬φ┐╦╩2╛▌═°í┐_2015.docx]

各位代表：

现在，我代表市人民政府向大会报告工作，请予审议，并请市政协各位委员和列席人员提出意见。

一、2015年和“十二五”工作回顾

去年是2008年以来宏观经济形势最为严峻的一年，面对前所未有的困难挑战，在自治区党委、政府和市委的坚强领导下，在市人大及其常委会、市政协的监督支持下，市人民政府牢牢把握经济社会发展主动权，及时调整发展思路，克难攻坚，真抓实干，较好地完成了市四届人大六次会议确定的各项目标任务。

（一）积极推动经济平稳较快发展。认真落实中央和自治区关于稳增长的系列决策部署，研究出台了简政放权、清理收费等42条稳增长措施，全市经济运行总体平稳、稳中有进、稳中有好。预计地区生产总值增长8%，财政收入增长10%，固定资产投资增长24%，城镇居民人均可支配收入增长6%，农民人均纯收入增长10%。

（二）狠抓产业提质增效。坚持强化“工业兴市、工业强市”发展思路，突出盘活存量，着力扩大增量，加强扶持服务企业，持续开展“市长服务企业接待日”活动，千方百计帮助企业稳定生产经营，有力推动了工业经济扩量提质。预计规模以上工业总产值增长13%，规模以上工业增加值增长8.3%；新增规模以上工业企业15家，新增年产值超亿元工业企业32家。现代农业稳步发展。预计农林牧渔业总产值增长4.2%。特色农业（核心）示范区建设扎实推进，新增家庭农场197家、农民专业合作社523家，农产品标准化生产基地7.1万亩；水稻生产综合机械化水平达74%；获全区富硒农产品认证10个。现代服务业加快发展。预计全年社会消费品零售总额增长8.2%。港口货物吞吐量5334万吨，增长1.8%。深入实施“荷城电商”行动计划，建成“电子商务进万村”标准化示范服务点131个。加强旅游业发展，接待游客总人数增长12%、旅游总收入增长20%。

（三）强力推进招商引资上项目。牢固树立招商引资是抓经济的基础和源头的理念，实行全民招商、重力招商，出台了“1+6”系列招商文件，成立了8个招商小分队，设立了4个招商联络点，主动“走出去”，积极“引进来”，招商引资取得了新突破。全市新引进项目176个，合同投资额579.5亿元，引进了投资50亿元的新能源汽车制造、投资10.5亿元的东盈纸业等一批大项目、好项目。加强项目攻坚和重点建设。全市规模以上主要建设项目471个，完成投资222.9亿元，其中新开工项目111个、竣工项目97个。列入“西江经济带项目建设三年攻坚计划”的80个重大项目，完成投资90亿元，有色金属新材料项目等3个项目实现竣工，市高铁站前广场、平南西江流域文化城一期工程等16个项目实现开工。

（四）统筹推进城乡基础设施建设。加强中心城区基础设施建设，青云大桥、罗泊湾大桥、中心城区至覃塘城市道路等重大城建项目加快推进，市体育中心、文化艺术中心、档案馆、中心医院等重点公共服务设施项目进展顺利。深入推进市容市貌、户外广告、交通秩序等专项整治，大力实施城市绿化、亮化、美化、彩化景观改造，城市面貌大为改观。加强农村基础设施建设，新建农村公路920公里，完成病险水库除险加固25座，新增或改善灌溉面积8万亩。扎实开展“美丽贵港·生态乡村”活动，完成自治区级示范村屯绿化253个，建设农村饮水安全项目306个。

（五）切实保障和改善民生。投入资金153亿元用于各项民生事业，新建了一批教育、卫生、文化等涉及群众切身利益的民生工程。社会保障不断强化。全年新增城镇就业1.55万人，城镇登记失业率2%，新增农村劳动力转移就业3.85万人。社会救助体系不断完善，社会保险覆盖率稳步提升，提高了城乡低保和五保户分散供养标准。加快推进安居工程建设，新建保障性住房和棚户区改造16364套，基本建成7849套；完成农村危房改造13800户。深入推进精准扶贫，全市4.2万贫困人口实现脱贫。深入推进“平安贵港”建设，认真抓好安全生产、食品药品安全监管、信访和矛盾纠纷调解工作，社会和谐稳定。

各位代表！2015年经济社会主要目标任务的基本完成，标志着我市“十二五”胜利收官。

——过去五年，是我市综合实力明显提升的五年。预计全市地区生产总值由2010年的545亿元提高到2015年的858.5亿元，年均增长7.5%；人均地区生产总值由12932元提高到20031元，年均增长6.7%；全社会固定资产投资由385.29亿元增加到745.9亿元，年均增长23%；财政收入由40.02亿元提高到72.7亿元，年均增长12.7%；城镇居民人均可支配收入由15531元提高到24657元，年均增长9.7%；农民人均纯收入由5289元提高到10045元，年均增长13.7%。

——过去五年，是我市经济结构不断优化的五年。坚持强化工业主导地位。规模以上工业总产值由2010年的470.3亿元增加到901亿元，年均增长14.4%；规模以上工业增加值由2010年的168.7亿元增加到270亿元，年均增长8.8%；预计年产值超亿元工业企业达200家，比2010年增加1倍。节能成效明显，预计规模以上万元工业增加值能耗比2010年下降26%。巩固农业基础地位。粮食生产连年丰收，主要农产品增产增效；现代特色农业（核心）示范区创建成效显著，“荷美覃塘”现代农业特色示范区与亚计山生态养殖特色示范区被评为广西省级特色农业示范区；新型农业经营主体快速发展，市级以上农业龙头企业达121家，比2010年增加了74家，农民专业合作社达1942家，比2010年增加了1207家。加快发展服务业。“十二五”时期，社会消费品零售总额累计完成1600.6亿元，年均增长13.2%。旅游接待总人数年均增长17.8%，旅游总收入年均增长29.9%；西山景区被评为“广西十佳景区”，覃塘“荷田水乡”入选全国休闲农业与乡村旅游示范点。港口货物吞吐量年均增长7%，金融、保险、电子商务等服务业稳步发展。

——过去五年，是我市城乡面貌显著变化的五年。累计完成固定资产投资2568.2亿元。城市规模扩大、功能提升。中心城区建成面积由2010年的51.1平方公里增加到64.5平方公里，城区新增绿地102.4公顷，人均新增绿地面积2平方米。新建中心城区迎宾大道北延段、建设路西延段等6条城市道路，打通了金田路、新华路等9条城区“断头路”，完成小街小巷改造180多条。市图书馆、博物馆、“天网”工程二期等一批重大城市配套设施建成，城区重点公共区域WIFI免费开放；完成马草江生态公园、东湖公园、南山公园改（扩）建工程，民族文化公园建成开放。加快推进各县城和重点镇建设，切实抓好城乡风貌和高铁沿线风貌改造。2015年全市城镇化率达47%，比2010年提高了6.8个百分点。加强城乡道路建设，南广高铁以及梧州至贵港、桂平至来宾（贵港段）高速公路全线贯通，新增等级以上公路1354公里，四级以上公路总里程突破6000公里，98.7%的行政村通达沥青或水泥路。组织实施西江经济带基础设施建设大会战，大藤峡水利枢纽、贵港二线船闸航运枢纽等11个项目实现开工。加强新农村建设。完成病险水库除险加固203座，完成中小型灌区渠道改造或硬化1300公里。建成农村饮水工程1089项，累计解决270万农村人口饮水安全问题。“清洁乡村”、“生态乡村”工作扎实推进，打造了25个市级生态乡村综合示范村。加强环保基础设施建设，市生活垃圾焚烧发电厂建成投入运营，建成乡镇垃圾中转站58座。

——过去五年，是我市改革开放深入推进的五年。深化以产权制度改革为重点的国有企业改制重组，推动经营性国有资产集中统一监管，实现国有资本及权益连续5年增长。扎实推进“营改增”、商事制度等改革，促进了市场主体快速发展，累计发展企业1.3万家。稳步推进行政审批制度改革，共取消、下放、调整行政审批336项。优化行政审批流程，加强电子政务建设。市、县、乡、村四级政务服务体系实现全覆盖，政务服务水平进入全区先进行列。深入推进财政管理体制改革，综合理财能力进一步提升。全面完成新一轮政府机构改革、事业单位分类改革和市不动产统一登记相关工作。县级公立医院综合改革试点平稳开展，城乡大病保险工作全面铺开。水利、林权、供销社等农村综合改革稳步实施。招商引资成效显著。“十二五”时期，累计引进项目1247个，合同投资额1268亿元，累计到位资金961.8亿元。对外贸易进一步扩大，外贸进出口总额累计13.5亿美元，年均增长13%；累计利用外资2.9亿美元，年均增长20.2%。对外友好交流更加密切，与韩国首尔特别市九老区、菲律宾三宝颜市建立了友好城市关系。

——过去五年，是我市社会事业全面进步的五年。坚持教育优先发展，学前教育“三年行动计划”一期全面完成，农村学校办学条件不断改善，提前一年实现自治区示范性普通高中县域全覆盖。以“民办公助”为模式的民办教育改革试点工作扎实推进。累计发放各类助学金11亿元，惠及学生160多万人次。深入实施创新驱动战略，累计获得省级以上重大科技成果10项；科技进步贡献率43.5%，比2010年提高了5.1个百分点。文化事业稳步发展，建成村级公共服务中心452个、农家书屋1134个，村村通广播电视综合覆盖率达98.2%；全市公共文化基础设施场所全部免费开放；“和为贵”文化品牌影响力不断增强。卫生事业加快发展，政府办基层医疗卫生机构实现国家基本药物制度全覆盖，新型农村合作医疗制度覆盖全市，农民平均参合率达到99%。诚信计生不断深化，人口自然增长率控制在9‰以内。国防动员、民兵预备役建设和双拥工作不断加强，荣获“全国双拥模范城”七连冠、“自治区双拥模范城”八连冠。残疾人、老年人和妇女儿童权益得到保护，统计、体育、气象、地震、地方志、民族宗教、对台事务等各项工作取得新成绩。

——过去五年，是我市民生保障明显提升的五年。累计投入民生资金569亿元，占财政支出的80.54%。社会保障和就业工作力度加大。五年新增城镇就业人数8.6万人，转移农村劳动力就业21.4万人，城镇登记失业率控制在4.3%以内。五项保险参保人数扩大到262.8万人次，社会保险制度实现了全覆盖。解决了符合条件的原民办教师、代课人员、被征地农民以及村干部等群体的养老保险问题，解决了市辖三区机关事业单位职工与市级“同城同酬”问题，提高了桂平市和平南县机关事业单位职工津补贴标准，提高了乡镇机关事业单位工作人员工作补贴。城乡居民医保待遇、企业退休人员基本养老金大幅提高。社会救助体系得到加强，城乡低保实现应保尽保。养老服务水平不断提高，建成五保村360个、农村幸福院255个，给80周岁以上高龄老人发放了生活津贴。累计建成保障性住房和棚户区改造住房24569套（户），发放租赁补贴14931户次，有力缓解了城乡中低收入群体以及乡镇中小学教师、卫生院和乡镇政府干部职工住房困难问题。扶贫工作扎实推进，累计减少贫困人口24万。环保工作成效明显，空气质量总体良好，主要江河段面水质达标率100%，城区生活污水处理率85%以上。应急管理体系建设、食品药品监管得到加强。“平安贵港”建设力度加大，全面完成“六五”普法教育，矛盾纠纷调解和信访工作成效明显。安全生产形势稳定。

——过去五年，是政府自身建设不断提升的五年。我们高度重视政府自身建设，认真贯彻落实中央“八项规定”，深入开展党的群众路线教育实践活动和“三严三实”专题教育，集中解决了一批群众反映强烈的“四风”问题，制定或修订了一批工作制度，有力提升了行政效能。全面推进依法治市，自觉接受人大、政协和社会监督，人大代表建议和政协提案办结率100%。推进政务公开和政府信息公开，强化行政监察和审计监督，深入推进反腐倡廉工作，制度执行力和约束力得到了增强。

各位代表！五年的成就极不寻常，五年的奋斗饱含艰辛。这是自治区党委、政府和市委正确领导、科学决策的结果，是市人大和市政协监督支持的结果，是全市上下团结拼搏、扎实苦干的结果。在此，我代表市人民政府，向全市人民，向各民主党派、工商联、无党派人士、人民团体，向中区直驻贵单位和驻贵部队、武警官兵，向所有关心支持贵港发展的各界人士、各位朋友，致以崇高的敬意和衷心的感谢！

回顾过去的五年工作，我们深深体会到，要把贵港的改革发展推向前进，必须坚持发展第一要务，突出产业发展这个关键，不断壮大工业经济，优化产业结构，提高质量效益，增强经济发展的协调性和持续性；必须坚持把保障和改善民生放在首位，深入推进城乡统筹，让改革发展成果惠及广大群众；必须大力发扬改革创新和求真务实精神，勇于探索解决新问题，善于统筹全局抓落实，努力提高各项工作成效。这些经验是指导我们今后做好各项工作的宝贵财富。

在肯定成绩的同时，我们也清醒地认识到，受宏观环境影响和自身基础条件限制，我市经济社会发展还存在不少困难和问题。主要是：经济总量小，人均水平低，财力薄弱，与全国、全区同步全面建成小康社会任务艰巨；经济结构不合理，工业规模小，农业现代化和城镇化水平不高；在工业化初期进入新常态，新的增长点不多，创新能力不足，实现经济持续稳定增长压力巨大；民生改善任务依然艰巨，公共服务水平与人民期盼差距较大，生态改善和环境保护更加紧迫。同时，政府自身建设仍需加强，政府职能和干部作风尚需加快转变。对此，我们将高度重视，主动作为，采取有效措施认真加以解决。

二、“十三五”时期的主要目标和任务

“十三五”时期，是全面建成小康社会的决胜阶段，也是我市适应新常态、推动新发展、打赢经济“翻身仗”的关键时期。根据市委关于国民经济和社会发展第十三个五年规划的建议，市政府制定了《国民经济和社会发展第十三个五年规划纲要（草案）》，提交大会审议。

《纲要（草案）》明确，“十三五”时期我市经济社会发展的总体要求是：高举中国特色社会主义伟大旗帜，全面贯彻党的十八大和十八届三中、四中、五中全会精神，以马克思列宁主义、毛泽东思想、邓小平理论、“三个代表”重要思想、科学发展观为指导，深入贯彻习近平总书记系列重要讲话精神，坚持发展为第一要务，以提高发展质量和效益为中心，以建设西江流域核心港口城市为目标，牢牢把握“工业兴市、工业强市”的发展主基调，坚持创新、协调、绿色、开放、共享发展。全面实施创新驱动、东进融合、港产城互动三大战略，着力创新区域发展与合作方式，创建经济发展战略平台，创新招商引资方式，创新市场经营模式，有效转变发展方式，推进发展顶层设计，增强我市发展优势。坚持稳中求进、进中求好，用发展新理念新思路引领我市西江经济带开发建设，全面统筹经济、社会、文化、生态建设和协调发展，为实现贵港全面振兴和时代新崛起，实现全面建成小康社会奠定坚实基础。

根据以上要求，综合研判我市经济社会发展的趋势和条件，“十三五”时期的目标任务是：

——经济保持较快增长。地区生产总值保持年均9%的较高增长速度，到2020年，实现地区生产总值和城乡居民收入比2010年翻一番以上，经济增长质量和效益明显提高，努力实现经济、社会、生态协调发展。

——结构调整取得重大进展。转变发展方式实现重大突破，东进融合与招商引资成效明显，工业结构不断优化，优势产业发展提速，产业规模快速扩大，工业主导优势更加明显。市场建设步伐加快，商贸、物流、旅游等现代服务业以及农业产业化实现突破性发展。城镇化与工业化发展协调同步，综合实力大幅提升。

——城乡居民生活水平不断改善。实现城乡居民收入与经济同步增长，社会就业面不断拓展，城乡居民的基本公共服务体系实现全覆盖，国家现行标准下农村贫困人口实现脱贫。

——开放合作不断深化。构建宽领域多层次对外开放合作平台，全面融入粤港澳战略取得重大进展，跨区域合作成效明显，政府职能和治理方式转变加快，创新发展理念成为社会共识。

——生态文明建设成效凸显。耕地保有量保持稳定，环境保护和生态治理成效明显，节能减排降耗实现自治区下达的目标任务，森林覆盖率保持稳定，森林质量、森林生态功能稳步提高。

为实现上述目标任务，我们必须坚定不移地推进跨越发展，始终把发展作为第一要务，促进全市经济规模效益同步增长，提升经济总量占全区的比重，增强综合实力和辐射带动力；必须坚定不移地推进转型发展，进一步强化“工业兴市、工业强市”发展思路，加快产业结构调整，逐步构建以现代农业为基础、战略性新兴产业为先导、先进制造业和现代服务业为支撑的现代产业体系，推动增长模式向资源节约型、环境友好型转变；必须坚定不移地推进协调发展，统筹城乡规划和建设，加速推进新型城镇化，扎实推进新农村建设，促进城乡公共资源共建共享，逐步缩小城乡差距；必须坚定不移地推进创新发展，大力推进思想观念、体制机制、方式方法创新，敢闯敢试、敢为人先，使创新成为引领发展的第一动力和干部群众的自觉行动；必须坚定不移地推进和谐发展，更加注重解决民生问题，更加注重发展社会事业，更加注重社会公平正义，促进改革发展稳定相协调，努力增进人民福祉。

三、2016年工作安排

2016年是实施“十三五”规划的开局之年，也是地级贵港市成立20周年，做好今年的工作，对我市在新起点上实现发展新跨越具有非同寻常的意义。我们将认真贯彻落实中央提出的去产能、去库存、去杠杆、降成本、补短板“五大任务”，自治区关于深入实施创新驱动、开放带动、双核驱动、绿色发展“四大战略”以及全面启动基础设施建设、产业转型升级和脱贫“三大攻坚战”，按照市委提出“工业大发展、项目大兑现、服务大提速、城市大变化、县域经济大突破、扶贫攻坚大成就”的目标要求，真抓实干，奋勇拼搏，努力为“十三五”开好局、起好步。今年经济社会发展的主要预期目标是：全市地区生产总值增长7.5%以上，财政收入增长8.5%，固定资产投资增长20%，规模以上工业增加值增长9%，社会消费品零售总额增长7.5%，城乡居民人均可支配收入增长8%。

围绕实现上述目标，我们将突出抓好以下重点工作：

（一）深入开展“工业提质年”活动，着力提升工业经济实力。

推动传统产业提档升级。以高端化、高新化、“两型”化、规模化为目标，支持现有企业技术改造和设备更新，提升自主创新能力，推动建材、冶金、化工、纺织服装与羽绒、造纸与竹木材加工、农产品食品加工、电力等传统优势产业提质升级。重点推进西江重工多用途货船双燃料动力、贵糖异地搬迁、华洋矿源扩建等一批技改项目，加快粤桂（贵港）热电联产循环经济产业园、钢材加工贸易物流园、有色金属新材料产业园等特色园区建设，促进产业链横向拓展、纵向延伸。稳妥化解产能过剩，积极推进企业兼并重组，淘汰落后产能。

大力发展战略性新兴产业。以装备和产品智能化及绿色低碳为发展方向，选择有一定基础、具有比较优势的领域进行高位谋划，强化政策支持，加快集群集聚。重点培育发展先进装备制造、新材料、新能源及电动汽车、电子信息、生物医药、节能环保等新兴产业。力争全面启动华奥新能源汽车产业园、低速电动车等项目建设，着力打造汽车制造产业集群。

加快产业园区建设。以现有园区为依托，坚持政府主导、市场运作，着力完善园区基础设施，增强园区承载能力。严格执行园区用地投资强度、环保等控制标准，推进工业地产和多层标准厂房建设，促进园区集约节约用地。

（二）深入开展“项目落地年”活动，进一步增强经济发展后劲。

加大招商引资力度。继续强化县域招商、部门招商、小分队招商、驻点招商和委托招商，严格执行以项目签约率、项目投资额、落地开工率为核心指标的考核奖惩办法。完善招商优惠政策和招商机制，整合招商力量，加快引进一批科技含量高、成长性强的项目。力争年内市产业园区落户投资超亿元项目13个以上，每个县市区各引进超亿元项目6个以上，全市完成招商引资实际到位资金300亿元以上。

切实加强重点项目建设。健全市领导联系重点项目推进机制，强化跟踪督查和考核激励机制，切实提高项目开工率和竣工率。确保年内上亿国际汽车贸易博览城、东盈纸业等一批重大项目开工建设，加快推进永泰仓储粮糖物流、乳泉井异地技改搬迁、港龙皮革城一期、永源生物科技园等在建重点项目建设，全力推进大藤峡水利枢纽、贵港航运枢纽二线船闸等西江经济带基础设施建设大会战项目，积极配合推进在建的县县通高速公路项目建设。

破解项目瓶颈制约。完善重点项目建设例会、联审联批、目标管理等推进机制，深入开展征地拆迁攻坚战，下决心解决项目落地难问题。加大存量用地盘活力度，依法处置闲置土地。拓宽融资渠道，积极向上争取差别化信贷政策，创新项目融资抵押和担保方式。加强重点项目政银企对接，引导金融机构加大项目信贷支持力度。积极推广PPP模式（政府和社会资本合作模式），加快基础设施、公用事业等领域项目建设。

（三）深入开展“企业服务年”活动，着力营造良好的发展环境。

积极服务市场主体。完善和落实扶持企业发展政策，降低企业的制度性交易成本，清理各种不合理收费，规范中介服务行为，进一步减轻企业和创业者税费负担。深化企业注册登记全程电子化等商事制度改革，促进全民创业、大众创新。强化项目帮办、绿色通道、预约服务、限时办结等便利化服务，严肃查处“吃拿卡要”行为，切实畅通企业发展“中梗阻”。

完善服务企业长效机制。继续定期开展“市长服务企业接待日”活动，建立服务企业联席会议制度和领导联系重点企业制度，强化职能部门协作和服务扶持，及时解决企业生产经营中存在的突出矛盾和问题。完善经济运行监测体系，定期会商研判经济形势，强化要素保障协调服务，及时有效应对经济波动。

（四）深入开展“城市建设管理年”活动，努力推进城市扩容提质。

完善城乡发展规划。创新城市规划理念和方法，加强对城市空间立体性、平面协调性、风貌整体性、文脉延续性等方面的规划和管控，统筹推进“多规合一”。年内编制完成城市近期建设规划，完善城市总体规划和专项规划，抓好重要街区天际线规划设计和骑楼街规划设计，修编乡镇总体规划和控制性详细规划，继续开展村庄建设规划工作，优化城乡空间布局。

加大城市建设力度。围绕提升中心城区综合服务功能，全力推动青云大桥、罗泊湾大桥、市高铁站前广场、贵港园博园、中心城区至覃塘城市道路、解放路下穿铁路工程、同济大道下穿铁路工程、城区道路白改黑工程和江南大道东段拓宽改造等重大项目建设。建成东环路，打通主城区城市环路。加快郁江北岸堤路园综合治理工程建设。抓好南山公园基础设施改造，推进堤路园、震塘和南江社区棚户区改造。规划建设一批特色街、精品路和标志性建筑，积极推进城市主题街区、新型社区建设，加强旧城区历史文化遗址的保护与开发。抓好4个城区出入口和迎宾大道、中山路、金港大道等重点街道景观绿化、亮化、美化、彩化改造，进一步提升城市形象。加大城市公交投入，增加公交线路，延长运行时间。积极推进县城和重点镇的路网、给排水、污水处理设施建设，增强城镇的辐射带动力；实施户籍、社保和公共服务等配套改革，有序推进农村人口向城镇流动和农业转移人口市民化，年内城镇化率达49%以上。

提高城市管理水平。完善城市治理体系，提高城市治理能力，着力解决城市病等问题。推进智慧城市建设，促进城市管理即时化、精细化、高效化。加强城市管线、环卫、公交等公共设施日常管护，保障各项设施正常运转。加大城市综合执法力度，加强城市卫生保洁和市容秩序管理。推动文明城市创建，强化市民文明卫生素质养成教育，实现城市环境美与人文美的协调统一。继续加大打击“双违”力度，不断规范建筑秩序。

着力改善人居环境。加强落实机动车尾气排放检测和环保标志管理制度，完善重污染天气监测预警体系和应急预案，积极推进大气污染防控，进一步提升城市空气质量。实行最严格水资源管理制度，加强饮用水水源地环境保护和流域水环境综合整治，加快推进中心城区城东和乡镇污水处理厂建设，深化工业污染防治，进一步改善水环境质量。推进建筑垃圾、生活垃圾资源化利用，加快规划建设桂平、平南垃圾焚烧发电厂。开展农村环境集中连片整治，加强农村污染源治理，继续推进规模化禽畜养殖污染治理工作。以创建国家森林城市为契机，加大造林绿化力度，年内完成造林6万亩，森林覆盖率达到46.35%，城市绿化覆盖率达到40.46%。

（五）深入开展“县域经济突破年”活动，着力推进区域协调发展。

加快打造特色产业。支持各县市区立足本地资源禀赋，明确产业重点，突出主攻方向，彰显区域特色。坚持分类指导，实行错位发展、差别竞争，重点支持桂平市加快发展生物化工和食品加工业，推动旅游业做大做强；支持平南县提升建材产业链，大力发展电子信息、生物科技等新兴产业；鼓励港北区大力发展城郊经济，加快发展绿色生态产业和休闲旅游业；支持港南区做大做强木业和羽绒加工业；支持覃塘区巩固发展水泥建材、板材加工等优势产业，加快发展新能源和船舶修造业，推动现代农业和乡村休闲农业融合发展。支持县市区抓好产业园区建设，引导各类要素向园区集中，促进关联产业聚集，实现错位发展。

充分发挥县市区的主观能动性。全面落实简政放权政策，加大支持、指导力度，及时帮助县市区解决发展中的实际困难和问题，让县市区有更大的空间创造性地开展工作。完善县域经济综合考核体系，大力营造比学赶超、竞相发展的良好氛围。

（六）深入开展“精准扶贫攻坚年”活动，扎实推进精准扶贫开发。

强化分类精准扶贫。大力实施“五年攻坚战、共圆小康梦”脱贫攻坚行动，加大资金、政策、工作等投入力度，切实做到结对帮扶、教育扶智、产业富农、农村危房改造、基础设施建设、政策扶持到村到户，力争年内实现7.5万贫困人口脱贫。全面落实学生资助政策，年内实现家庭经济困难学生资助全覆盖，确保学生不因家庭经济贫困而上不起学；每个贫困村培育发展1-2个特色主导产业，促进贫困户稳步增收，贫困地区农民人均纯收入增长13%以上；有计划、分步骤推动养老、助残、医疗、低保等各项农村社会保障制度向贫困人口倾斜，落实保障政策覆盖。

强化扶贫资源整合。全面整合涉农资金，加大对贫困地区倾斜力度，年内投入4000万元用于贫困地区基础设施建设，修建村屯道路220公里以上。创新金融扶贫机制，建立扶贫产业投资基金，完善扶贫贷款贴息政策，引导和鼓励商业性银行机构创新金融产品和服务方式，加大对贫困户的信贷投放力度。积极探索政策性农业保险与“三农”小额贷款有机结合，为贫困地区农业增产、农民增收提供更多更好的金融保险服务。坚持社会帮扶和自力更生相结合，引导各类企业、社会组织和个人以各种形式参与扶贫攻坚，形成扶贫开发强大合力。

强化脱贫攻坚责任落实。严格党政“一把手”负总责的脱贫攻坚责任制，健全市级统筹、县为主体、乡村落实、部门配合的工作机制和扶贫开发责任体系，全面落实扶贫责任、权力、资金、任务“四到县”制度。深化领导联系帮扶、机关单位对口帮扶、干部驻村结对帮扶等制度，确保每个贫困村都有帮扶工作组，每个贫困户都有帮扶责任人，实现贫困村、贫困户结对帮扶全覆盖。

（七）加强“三农”工作，加快构建现代高效农业生产体系。

着力发展现代农业。坚持特色发展，在稳定粮食生产的同时，进一步优化农业区域布局，扩大中药材、水果等特色农业的种植规模及加工，抓好“双高”糖料蔗示范基地和常年保障性蔬菜基地建设，促进农产品提质增效。坚持标准化生产，加快推进桂平市粮食产业全程社会化服务示范区、港北区富硒优质稻示范区等12个现代农业示范区建设，深入开展畜禽养殖和水产健康养殖标准化示范创建工作，促进农业规模化、标准化、集约化发展。实施农业品牌战略，重点抓好富硒优质稻、富硒茶等生产示范基地建设，规划建设贵港富硒食品加工区，做大富硒品牌。积极推动农业产业化发展，引导土地有序流转，发展适度规模经营，年内培育发展市级以上农业龙头企业10家、示范性农民专业合作社20个。培育多元化市场流通主体，搭建农超、农商、电商对接平台，拓宽农产品销售渠道。充分挖掘和拓展农业功能，推进休闲农业与乡村生态旅游开发，重点抓好港北区北环生态农业景观带、覃塘区“荷美覃塘”莲藕产业（核心）示范区、港南区“四季花田”示范区和桂平市金田淮山产业（核心）示范区建设，培育农民增收新亮点。

着力改善农村生产生活条件。加强农田水利设施建设，年内实施病险水库除险加固工程5座、中小河流治理项目10个，恢复或改善灌溉面积4万亩，完成覃塘区整县推进高标准基本农田项目建设，开工建设桂平市整县推进高标准基本农田项目。继续推进移民新村建设，年内实施水库移民新村基础设施项目86个。加强“美丽贵港·生态乡村”建设，年内实施自治区级绿化示范村屯257个，农村饮水净化示范工程20个，硬化村屯道路300公里。

（八）立足自身优势，着力发展现代服务业。

大力发展商贸物流业。围绕打造西江流域商贸物流中心，加快美家居国际博览城、西江华轩物流中心等商贸流通项目建设，逐步完善商贸物流配套设施。规划建设贵港现代物流园、汽车交易贸易等服务业集聚区。支持流通业态创新，着力推行连锁经营、电子商务等现代商贸方式，改造提升传统商贸业。继续实施“荷城电商”计划，大力推进“电子商务进农村活动”，促进农村现代化流通体系建设。重视发展港口运输业，年内货物吞吐量增长6%以上。

加快发展旅游业。重点推进太平天国金田起义遗址红色旅游景区、西山泉国际旅游文化综合区、北帝山旅游区、铜鼓湾温泉生态度假村等重大旅游项目建设，提升旅游景区景点档次。继续推进旅游名县名镇名村建设，加强特色民居改造。加大旅游资源的策划包装和宣传推介力度，围绕宗教文化游、生态乡村游、休闲度假养生游，着力打造一批精品旅游路线。年内接待游客人数增长10%，旅游总收入增长15%。

培育发展新兴服务业。鼓励发展评估评价、检验检测、科技等生产性服务业，积极发展教育培训、家政服务、健康养老、休闲养生等生活性服务业。提高金融创新和服务水平。进一步优化金融生态环境，吸引更多金融机构在我市设立分支机构；鼓励金融产品创新，扩大信贷投放；支持企业上市、发行债券，扩大直接融资规模；进一步完善中小企业金融担保服务体系，帮助解决中小企业融资难问题。

（九）坚持改革开放，着力增强发展活力。

深化重点领域改革。深化行政审批制度改革，继续压缩、取消、下放市级行政审批事项，优化审批流程，缩短审批时限；积极探索和推动行政审批局机构组建工作，启动规划建设“市民中心”。深化国企国资改革，完善国资监管方式，提高国有资本运营效率。加快财税体制改革，完善政府预算体系，大力推行预算公开；加强政府债务管理，将政府性债务分类纳入全口径预算管理。进一步规范各类融资行为，防范化解金融风险。积极推进县级公立医院综合改革，支持社会资本举办非营利性医疗机构。深化民生社会事业改革，全面推进机关事业单位养老保险制度改革，扎实推进养老服务、社会救助、社区建设、社会组织建设等领域改革创新。加快农村综合改革，继续抓好农村土地承包经营权确权登记颁证试点工作。有序推进党政机关公务用车、国有林场、环保、城管、供销社等方面的改革。

积极扩大开放合作。对接“一带一路”发展战略，加强与珠江—西江经济带城市合作，主动融入黔粤桂高铁经济带，积极承接发达地区加工贸易和产业转移。认真落实《梧玉贵区域一体化发展规划》。积极推进西江保税物流中心（B型）申报建设工作，加快电子口岸建设。用好用足国家和自治区外经贸发展资金，深入实施加工贸易倍增计划，鼓励支持外贸企业实施品牌战略，进一步开拓国际市场，促进外贸稳定增长。年内外贸进出口总额和实际利用外资均增长5%以上。继续密切与韩国首尔特别市九老区、菲律宾三宝颜市友好城市关系，加强产业、人文、旅游等方面的交流合作。

（十）坚持共享发展，不断增进民生福祉。

突出抓好创业就业。鼓励支持初次创业和小微企业发展，激发创业创新活力。做好高校毕业生、农村转移劳动力、退役军人、就业困难人员等重点群体就业工作，年内新增城镇就业1.3万人，城镇登记失业率控制在4.3%以内，农村劳动力转移就业3.6万人，动态消除城镇“零就业”家庭。落实好最低工资制度，健全欠薪整治机制，推进构建和谐劳动关系。

提高社会保障水平。加强城乡低保规范管理，适时提高企业退休人员养老金、城乡低保、五保供养标准。完善城镇基本医疗、工伤、失业保险市级统筹机制，加强社会保险扩面征缴工作，年内各项社会保险参保人数达265万人次以上。加强社会救助工作，加快市社会福利中心建设，广泛开展关爱留守人员活动。增强社会养老服务能力，抓好县市区养老服务聚集区建设，加强农村养老服务机构的规范管理，鼓励发展民办养老机构、社区养老服务机构及老年人日间照料中心。加强保障性安居工程建设，年内开工建设公共租赁住房4902套、棚户区改造房3040套。

坚持教育优先发展。加大教育投入，统筹推进各类学校协调发展。完善教育基础设施，年内中心城区新开工建设一所幼儿园、两所小学、一所初中，确保荷城一幼、荷城二小和三小、达开高中扩建工程建成并投入使用，加快解决学校“大班额”问题。大力发展学前教育，确保学前三年毛入园率达75%以上。进一步健全义务教育经费保障机制，优化学校布局规划，全面推进义务教育薄弱学校改造计划，加强教师队伍建设，改善师资队伍结构，促进义务教育县域均衡发展，确保九年义务教育巩固率达93%以上。加强薄弱普通高中建设，推进普通高中多样化、特色化发展，确保高中阶段毛入学率达87%以上。大力实施现代职业教育发展工程，加快西江职教园区规划建设，力争年内引进1-2所学校入园办学。加大民办教育扶持力度，推动民办教育健康发展。合理配置城乡教育资源，保障进城务工人员子女入学。

统筹发展其他社会事业。建立健全公共文化体育服务体系。充分发挥“和为贵”文化促进社会和谐的载体作用，大力弘扬尚和向善的社会风气；加快推进市文化艺术中心建设，年内市体育中心、档案馆竣工投入使用，新建村级公共服务中心90个；加强文化遗产和非物质文化遗产保护开发，传承和发展传统优秀文化；加快推进乡村全民健身设施建设，广泛开展全民健身运动，提高人民群众身体素质。深入实施创新驱动发展战略。全面启动贵港高新技术开发区创建工作，加快广西农业科技园、科技企业孵化器和众创空间建设，促进科技成果转化应用。积极发展卫生事业。加快完善全民医保体系，提高基层医疗机构诊疗水平，努力推进基本公共卫生服务均等化；加快发展中医药事业；加强重大疾病防控和重特大疾病救助；完善新农合市级统筹工作，年内新农合参保率保持95%以上。深化计划生育服务管理改革创新，认真落实“全面两孩”政策，综合治理出生人口性别比，促进人口长期均衡发展。深入推进民族团结进步创建活动，依法管理宗教事务。认真做好双拥、优抚安置和人防工作，加强国防动员和国防后备力量建设，推进军民融合发展。充分发挥工会、共青团、妇联等群团组织的作用，统筹推进统计、物价、地震、地方志、对台事务、质量监督等工作。

加强和创新社会治理。支持群团组织依法参与社会治理，发展专业社会工作、志愿服务和慈善事业。引导群众依法有序表达诉求，坚持领导干部接访和积案化解。健全矛盾纠纷调处机制，完善和推广人民调解协议司法确认机制。认真组织实施法制宣传教育第七个五年规划，做好法律援助和社区矫正工作。严格落实安全生产责任，严防重特大安全生产事故发生。健全应急管理体系，切实抓好地质、气象等灾害防治。深化“平安贵港”建设，加强社会治安综合治理，积极构建立体化治安防控体系，依法防范和惩治违法犯罪活动，保障人民生命财产安全，提升人民群众的安全感和满意度。

四、加强政府自身建设

新常态需要新作为，新要求需要新作风。做好今年各项工作，全力打好“经济翻身仗、城市主动提升仗、脱贫攻坚战、作风建设持久仗”，开创“十三五”发展新局面，各级政府及其工作人员肩负着重大责任。必须以开明开放的理念、创新创造的精神、敢抓敢管的作风，埋头苦干，勤勉尽责，不断提高政府的创新力、执行力和公信力，决不辜负全市人民的信任和期望。

坚持务实高效。健全决策部署、检查落实、监督反馈工作制度，以重点目标、重要工作、重大事项的落实为着力点，把每项工作的责任落实到各级各部门、落实到个人，强化督导，跟踪问效，严格考核，兑现奖惩。完善牵头部门责任制，明确联动协作单位责任分工，形成责任主体明确、责任分工明晰的部门责任体系。严格执行首问负责制、限时办结制和责任追究制，全面提升行政效能。

坚持开拓创新。加强学习研究，把握发展规律，努力克服“换挡焦虑”和“本领恐慌”。坚持深入基层，加强调查研究，了解群众真实意愿，倾听群众心声，使各项政策措施更加符合发展实际、符合群众利益。进一步解放思想、大胆创新，注重研究发展中的新情况、新问题，把工作重点放在培育壮大新兴产业上、放在统筹城乡发展上、放在改善民计民生上，善于以创新的思路寻求事业的新突破，以担当的精神开创工作新局面。

坚持依法行政。稳步推进法治政府建设，深入实施法治教育工程、权力规范工程、依法决策工程、阳光政务工程和法治评价工程等“五大工程”，严格规范行政权力运行。坚持以法治引领和规范各项工作，用法治的思维解决社会热点难点问题。完善重大行政决策法治化程序，落实公众参与、专家论证、风险评估、合法性审查、集体讨论决定的决策机制。依法接受人大及其常委会法律和工作监督，主动接受政协民主监督，自觉接受舆论监督和群众监督。深入推进政务公开和政府信息公开，全面落实行政执法责任制，让权力在阳光下运行。

坚持清廉为政。严格落实廉政建设责任制和主体责任，强化“一岗双责”，把廉洁从政贯彻到政府工作各个环节。以权力“瘦身”为廉政“强身”，注重从源头上预防腐败。加强行政监察和审计监督，对公共资金、公共资源、国有资产严加监管。厉行勤俭节约，反对奢侈浪费，把有限的资金更多用于发展经济和改善民生。始终保持反腐高压态势，严肃查处发生在群众身边的“四风”和腐败问题，营造风清气正的良好氛围。

各位代表！站在历史新起点，面对人民新期待，应对经济新常态，我们深感责任重大、使命光荣。让我们在市委的坚强领导下，紧紧依靠全市人民，振奋精神，真抓实干，克难攻坚，为全面完成今年各项目标任务，不断开创贵港“十三五”时期经济社会发展新局面而努力奋斗！
